# HPV-related diseases in male patients: an underestimated conundrum

**DOI:** 10.1007/s40618-023-02192-3

**Published:** 2023-09-28

**Authors:** A. Garolla, A. Graziani, G. Grande, C. Ortolani, A. Ferlin

**Affiliations:** 1 Unit of Andrology and Reproductive Medicine, Department of Medicine, University of Padova, Padua, Italy; 2 Section of Otolaryngology, Department of Neurosciences, University of Padova, Padua, Italy

**Keywords:** HPV, HPV-related diseases, Head and neck cancer, Male HPV, Male infertility, Sperm parameters

## Abstract

**Purpose:**

Human papillomavirus (HPV) infection is the most common sexually transmitted disease, in males and females worldwide. While the role of HPV in female diseases is well known and largely studied, males have negligibly been included in these programs, also because the proportion of women suffering and dying from HPV-related diseases is much larger than men. The aim of this review is to focus on HPV-related diseases in male patients.

**Methods:**

We performed a literature analysis on the electronic database PubMed. We considered randomized trials, observational and retrospective studies, original articles having as topic the relationship between HPV male infection and the following items: oral, anal penile cancers, warts, condylomas, male infertility, altered sperm parameters, anti-sperm antibodies (ASA). We also included experimental in vitro studies focused on the effects of HPV infection on oocyte fertilization, blastocyst development, and trophoblastic cell invasiveness. In addition, studies describing the adjuvant administration of the HPV vaccination as a possible strategy to promote HPV clearance from semen in infected males were included.

**Results:**

Regarding head and neck HPV-related diseases, the most important non-neoplastic disease is recurrent respiratory papillomatosis (RRP). Regarding neoplastic diseases, the proportion of head and neck cancers attributable to HPV has increased dramatically worldwide. In addition, nowadays, it is thought that half of head and neck squamous cell carcinomas (HNSCCs) cases in the United States are caused by infection with high-risk HPV. HPV is noteworthy in andrological practice too. It was described as having a high HPV prevalence, ranging between 50 and 70%, in male penile shaft, glans penis/coronal sulcus, semen as well as in scrotal, perianal, and anal regions. Moreover, in male patients, HPV infection has been associated, among other diseases, with penile cancers. HPV semen infection has been reported in about 10% in men from the general population and about 16% in men with unexplained infertility, although these data seem widely underestimated according to clinical experience. In particular, HPV semen infection seems to be most related to asthenozoospermia and to anti-sperm antibodies (ASAs).

**Conclusions:**

HPV infection represents a health problem with a detrimental social and public impact. Despite this evidence, little has been done to date to widely promote vaccination among young males.

## Background

Human papillomavirus (HPV) infection is the most common sexually transmitted disease (STD) in males and females worldwide [1, 2].

It is estimated that the probability of infection with the virus is about 80% in females and 90% in males across their lifetime [3, 4]. The high prevalence of HPV infection in the general population is related to its contagiousness. Despite HPV being mainly transmitted through sexual activity, people can also be easily infected by skin-to skin contact [3].

HPVs belong to the family *Papillomaviridae* [3]. Papillomaviruses are a family of DNA viruses that infect the epithelium and mucosae and have a double-stranded, circular genome [3]. Based on the genomic sequence of L1, the gene encoding the principal capsid protein, scientists have identified over 200 subtypes of HPV, which are broadly categorized into high-risk and low-risk types [1, 3, 5, 6].

Low-risk HPV (LR-HPV) subtypes are thought to be responsible for skin, flat, or plantar warts [6]. Moreover, other LR-HPV-related diseases are oral and anogenital warts [6, 7] and condyloma acuminata [8]. The association with warts is so strong that HPV was first described as “human warts virus” [9]. Therefore, LR-HPV usually causes subclinical infections or benign papillomas [1].

Most frequently detected high-risk HPV (HR-HPV) subtypes include, among others, HPV-16, 18, 31, 33, 35, 39, 45, 51, 52, 56, 58, 59 [1]. Two of these, type 16 and 18, are responsible for the majority of HPV-related cancers, including cervical, anal, penile, and oropharyngeal cancers [4]. The strong evidence of HPV role in causing cancer has been stated by the International Agency for Research on Cancer (IARC) for cancers of the cervix uteri, penis, vulva, vagina, anus and oropharynx [2].

In fact, HPV has a prominent role in cancer etiology, since the virus is responsible for about 30% of all infectious agent-related cancers [7]. Worldwide, HR-HPV subtypes cause about 5% of all cancer cases, with an estimated number of infections of 570,000 in women and 60,000 in men each year [3].

Considering both males and females, the overall prevalence of HPV infection is about 40% of the general population, with variations based on the HPV type and the anatomical site of infection [2]. It has to be noted that the majority of infected women and men do not present with clinically significant signs or symptoms, or they may experience a transient infection [9]. The burden of HPV-associated diseases is greater in immunocompromised persons, such as those with human immunodeficiency virus (HIV) infection, those who have undergone transplant [5] or cancer treatments [10]. This evidence is so strong that the results of a recent study, evaluating the risk of anal HPV infection in kidney transplant recipients, might indicate that pretransplant HPV vaccination should be considered in patients undergoing transplant (in this case kidney transplant) [11].

Regarding HPV-related disease in females, cervical cancer is the 4th cause of cancer worldwide. HPV type 16 is responsible for 50% of cases, and, together with type 18, accounts for 70% of this condition [6]. Other studies reported that, in female patients, HPV is estimated to be the cause of 99% of cervical cancers, 90% of anal cancer, 65% vaginal cancers, 50% vulvar cancers, and 45–90% oropharyngeal cancers [6].

While the role of HPV in female diseases is well known and largely studied, due to many screening and research clinical programs, males have negligibly been included in these programs, also because the proportion of women suffering and dying from HPV-related diseases is much larger than men [7]. In males, the highest prevalence of becoming infected with a type of HPV was observed in HIV-infected men who have sex with other men [3]. More recently, studies on the clinical consequences of HPV infection have been extended to the heterosexual male population. In this context, the role of males in the transmission of HPV to women has been explored too [7]. In the last years, the issue has been greatly deepened and nowadays there are several data obtained regarding HPV infection and males available on the current literature. Nevertheless, sensibleness in male HPV infection and related diseases is still insufficient and less than that regarding female HPV infection [1, 9].

There is actually no accepted and validated test for screening HPV in males [7]. However, there is a general consensus on when diagnostic testing should be performed: (i) in case of a partner who tested positive HPV or has HPV-related diseases; (ii) in the presence of HPV-related clinical manifestations; (iii) in men who have sex with men; (iv) in males with idiopathic infertility; (v) in case of HIV infection [7, 12].

Both in females and males, HPV infection is usually transient, and the associated lesions have a high remission rate. However, persistent HPV infection with HR subtypes is associated with invasive carcinomas, especially among women and immunocompromised patients.

**Fig. 1:**
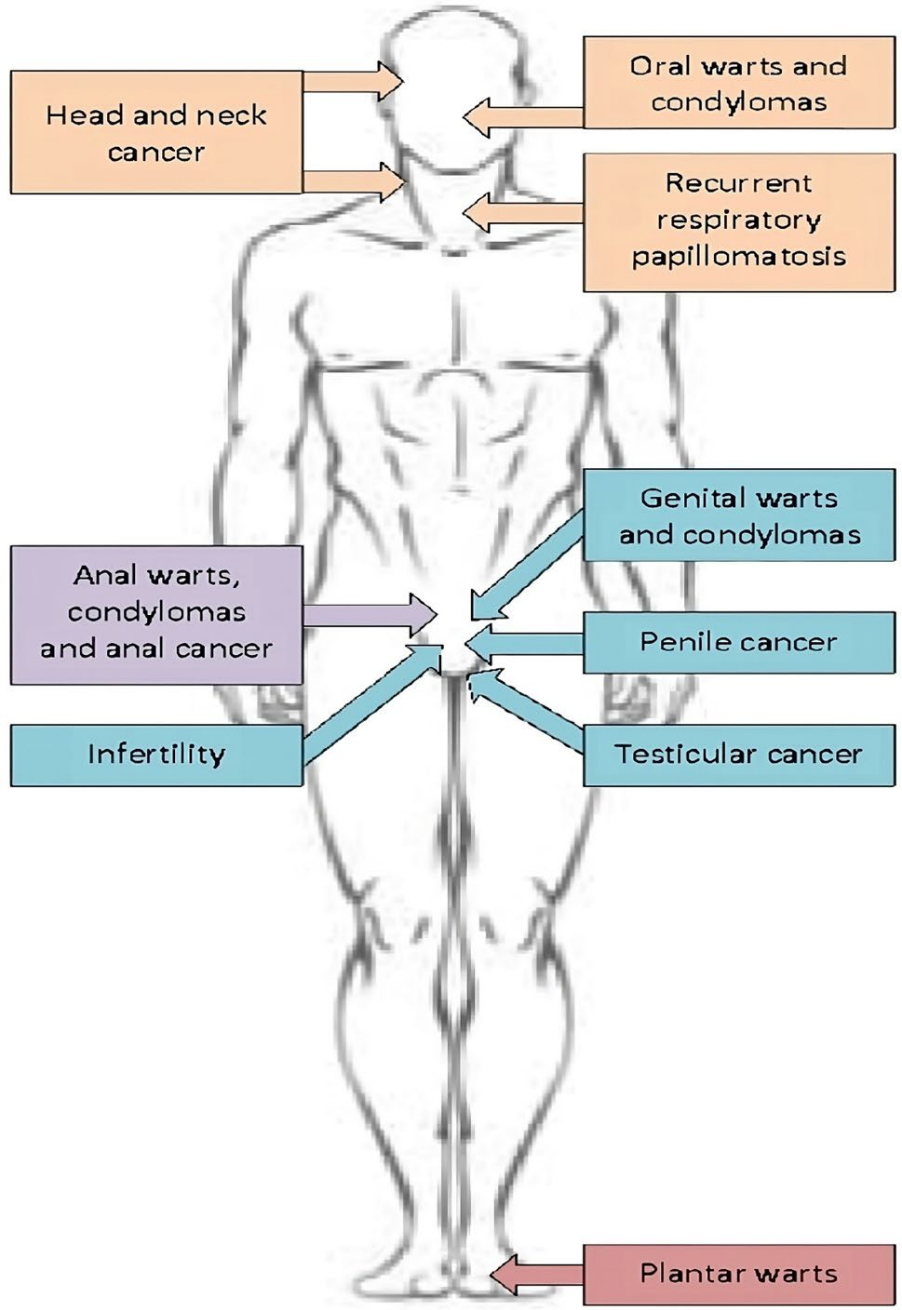
HPV-related diseases in males

The aim of this review is to focus on HPV-related diseases in male patients. In particular, we will focus on HPV-related head–neck diseases and andrological problems, as shown in Fig. 1.

## Methods

In order to review the recent literature on HPV-related diseases in males, we performed a literature analysis on the electronic database PubMed considering different time intervals: from 2020 to 2022 for head and neck diseases, from 2012 to 2022 for andrological diseases and from 2012 to 2022 for male infertility. Key terms included, respectively: “HPV and head and neck”, “HPV and male” and “HPV and male infertility”. We considered randomized trials, observational and retrospective studies, original articles having as topic the relationship between HPV male infection and the following items: oral, anal penile cancers, warts, condylomas, male infertility, altered sperm parameters, anti-sperm antibodies (ASA). We also included experimental in vitro studies focused on the effects of HPV infection on oocyte fertilization, blastocyst development, and trophoblastic cell invasiveness. In addition, studies describing the adjuvant administration of the HPV vaccination as a possible strategy to promote HPV clearance from semen in infected males were included.

### HPV in head and neck diseases

The history of HPV in head and neck sites starts in 1901 when the contagious transmission of warty lesions into the mouth via oral sex was described [13]. During the following decades, HPV was associated with development of laryngeal warts, koilocytotic atypia and head and neck cancer [13].

A higher number of HPV infections of the head and neck region have been reported in men compared to women. This is probably due to a high number of men giving oral sex to HPV-infected partners [14].

The most important non-neoplastic disease related to HPV in the head and neck region is recurrent respiratory papillomatosis (RRP). There are two clinical presentations of the disease—juvenile-onset RRP (JoRRP), when the condition occurs under 12 years of age, and adult-onset RRP (AoRRP) [15].

This disease is frequently associated with mucosal HPV-type infection, in particular HPV-6 and 11, and characterized by the growth of squamous papilloma in the airway epithelium [15, 16].

JoRRP is prevalent in sub-Saharan Africa. Transmission of HPV is believed to occur during birth from the mother as the fetus passes through an infected genital tract [15]. Laryngeal papillomas usually manifest with progressive hoarseness, stridor, or even severe airway obstruction [17]. There is currently no eradicative cure for JoRRP. Moreover, JoRRP is typical recurrent, thus patients require multiple surgical debulking procedures to maintain an airway patency [17].

AoRRP usually develops after 20 years of age, in the third and fourth decades of life, more commonly in men. In adults, HPV is transmitted sexually, through oral contact with infected external genitalia [18, 19]. Clinically, in adults, hoarseness is the most common finding [19]. In this form, the papillomas are often solitary, they do not usually spread, and recur less frequently than those seen in the juvenile form [19].

Regarding neoplastic diseases, while tobacco and alcohol were historically the main drivers of head and neck cancers, in the past several decades, the proportion of head and neck cancers attributable to HPV has increased dramatically worldwide [20, 21]. Head and neck cancers (HNC) represent about 4.8% of cancers. Ninety percent of HNC arises from squamous epithelial cells lining the oral cavity, pharynx, larynx, or, more rarely, the nasal cavity [14]. The incidence of HNC varies, depending on the anatomical region and geographical location. Nevertheless, it is certain that the incidence of HNC has increased by 36.5% over the past decade [20].

HPV has been hypothesized to play a role in the etiology of head and neck squamous cell carcinoma (HNSCC) since many decades ago, in particular as early as 1983 [22]. Kreimer et al. reported that HPV prevalence was 35.6% in oropharyngeal cancers, 23.5% in oral cancers and 24.0% in laryngeal cancers, whereas overall prevalence of HPV in HNSCC was estimated at 26% [23]. A more recent meta-analysis estimated that prevalence of HPV in oral, pharyngeal and laryngeal SCC was 34.5% [24]. Thus, during recent years, the incidence of HPV-related HNSCC has significantly increased [25]. Nowadays, it is thought that half of HNSCC cases in the United States are caused by infection with high-risk HPV [26].

A recent study showed that oral HPV infection is mostly sexually transmitted: the authors reported that oral HPV prevalence was more than eightfold higher among individuals who referred sexual intercourses vs no sexual activity [27]. Moreover, regarding HPV oral infection, a systematic review and meta-analysis [28] found that oral HPV incidence was threefold higher in HIV-positive men who have sex with men than HIV-negative men who have sex with men and that in this analysis there was no evidence that men who have sex with men are more at risk of oral HPV infection than heterosexual men.

Infection with high-risk HPV types drives tumorigenesis through expression of the oncoproteins E6 and E7 [25]. While E6 mainly acts binding p53 and causing its degradation, E7 enables the degradations of the tumor suppressor protein Rb [29].

HPV-16 and HPV-18 subtypes contribute to the majority (85%) of HPV-related HNSCC [14] while the remaining 15% of HPV-related HNSCC are mostly caused by HPV-33, HPV-35, HPV-52, HPV-45, HPV-39 and HPV-58 [14, 30].

The oropharynx is the most common site of head and neck cancer attributable to HPV. In particular, lesions develop in lympho-epithelial sites such as palatine tonsil and the base of the tongue [27]. It is believed that the crypts and irregular surface of the tonsils and lymphoid tissue in the base of the tongue create a favorable environment for HPV infection to persist [31]. This aspect appears to be most relevant for HPV persistence, increasing the risk of oropharyngeal squamous cell carcinoma (OSCC) development [32].

HPV infection is found in 20–60% of the OSCCs depending on the country [33]. HPV-induced OSCCs occur more often in non-smokers and are associated with more frequent nodal involvement [33]. Thus, the first symptom of HPV-related OSCCs is cervical swelling [32]. Furthermore, HPV-positive OSCC patients are often younger than HPV-negative OSCC patients [32].

As said above, the incidence of HPV-associated OPSCC has been increasing, particularly in younger age groups with no or very little tobacco exposure, and mostly in North America and northern Europe [20]. Moreover, OSCC is currently the most frequent HPV-driven cancer in the USA [20, 34], even more than cervical cancer in females [34].

An increasing incidence has been described for HPV-associated HNCs, in particular for OSCC, whereas the incidence of all other HNCs decreases in developed countries. This evidence is mainly due to the increasing prevalence of HPV in OSCC [32].

HPV oral infections occur more frequently in males [35]. Moreover, it is to remember that oropharyngeal cancer mostly occurs in male patients [32]. This evidence is noteworthy because sexual transmission is not the only route of transmission of HPV. In fact, other routes include fomites, fingers, mouth, skin contact and even self-inoculation [6].

As previously anticipated, HPV-positive OSCC comprises a distinct disease entity, with a different clinical and biological behavior [21, 36, 37]. Moreover, as said above, HPV-positive OSCCs present a better prognosis than HPV-negative OSCCs. This evidence may be due to the fact that HPV-positive OPSCCs tend to present with large metastases in the cervical lymph nodes, with a clinically and radiologically occult primary [37], allowing an earlier diagnosis and, therefore, a different prognosis.

The presence of HPV in cancer tissues in associated with a higher frequency of intra-tumoral B cells presence, whereas in HPV-negative cancers, there is a higher frequency of dysfunctional CD8+ T cells [26].

Furthermore, the evaluation of HPV status has been incorporated in treatment guidelines due to a major impact in prognosis which is also reflected on the new Tumor Node Metastasis (TNM) staging for HPV+ disease [38]. In particular, regarding OSCC, patients with HPV-associated disease have been shown to have a substantially better prognosis and overall survival than patients without HPV infection [39, 40]. This is well supported by the guidelines and by the clinical experience obtained in our head and neck surgery unit.

The mortality risk of patients with HPV-associated OSCCs seems to be about 50% lower in comparison to patients with HPV-negative OSCC, mostly due to improved locoregional control and increased radiation sensitivity. In addition, second primary tumors are significantly less frequent in patients with HPV OSCC [32].

Therefore, HPV-related biomarkers can be useful for OSCC’s early diagnosis, post-treatment surveillance and recurrence [40]. In particular, p16^INK4a^ over-expression in affected tissues is a marker of HPV involvement, widely used as an implemented technique in the clinical setting [41].

Finally, HPV has recently been associated to the middle ear squamous cell carcinoma (MESCC), a very uncommon head and neck cancer subtype [42]. Moreover, it was seen how a high percentage of patients with MESCC presented with otorrhea, although MESCC prognosis does not seem to be influenced by the presence/absence of HPV [42]. In Table 1, related articles published in the interval time January 2020–December 2022 are reported.

**Table 1:** Articles regarding main HPV-related head and neck diseases, published in the period January 2020–December 2022

References	HPV-associated head and neck diseases in men
[43]	About 70% of HNSCC, especially in deep crypts in the palatine and lingual tonsils; different clinic, therapy and prognosis in HPV-negative and positive HNSCCs
[44]	HPV-16 accounts for 86.7% OPSCC, 68.2% for oral squamous cells cancers and 69.2% for laryngeal squamous cells cancers
[33]	20–60% of OPSCCs; HPV-OPSCCs have a better prognosis than non-HPV-OPSCCs, with a better sensitivity to radiations and a better overall survival
[45]	21–26% HPV prevalence in sinonasal squamous cell carcinoma
[46]	HPV-HNSCC often presents a better prognosis than negative HPV-HNSCC, with a better response to the treatment and with wild type TP53 and low EGFR expression
[36]	90% of OPSCCs are caused by HPV-16
[20]	62% of male OPSCC patients and 56% of female OPSCC patients have HPV-positive tumors
[47]	About 50% of OPSCCs in western countries are HPV-positive, with higher rates in the United States and Scandinavia, and lower rates in Southern Europe
[48]	RRP is a rare neoplasm of the larynx cause by HPV
[37]	HPV-OPSCC has a better prognosis than non-HPV-OPSCC
[49]	80–90% of OPSCCs cases are due to HPV-16 infection and would be preventable with HPV vaccines
[50]	HPV-related OPSCC has a better survival outcome than HPV-negative cases
[51]	25–65% of OPSCCs worldwide are attributable to HPV infection
[52]	38–80% of OPSCCs
[53]	HPV infection in OSCC ranges between 6 and 58% and HPV-16 is the most frequent genotype detected, up to 88%
[54]	A possible role of HPV in the pathogenesis of sinonasal inverted papilloma
[55]	7.4% of oral cavity and lip cancer, 5.7% of laryngeal cancer, 7.9% of nasopharyngeal cancer, 24.9% of oropharyngeal cancer, 3.9% of hypopharyngeal cancer
[56]	30% of the head and neck squamous cells papillomas: laryngeal papillomas (76–94%), oral (27–48%), sinonasal (25–40%), oropharyngeal papillomas (6–7%) and esophageal (11–57%)
[57]	HPV-OPSCC accounts for 25–30% of all HNSCC cases and about 70% of OPSCCs are caused by HPV
[58]	HPV-16 is responsible for approximately 10% of all HNSCC and over 50% of tonsillar squamous cell carcinomas
[59]	22–47% of OPSCCs, with more than 90% of these being caused by HPV-16
[31]	The incidence of HPV- oropharyngeal cancer has surpassed HPV cervical cancer; HPV is the cause of 18–22.4% of OPSCC
[60]	OPSCC is the first cause of HPV-related cancer in the US
[61]	Emerging prospects for the beneficial use of therapeutic vaccines, as well as for targeted, molecular-based therapies for HPV + OPSCC
[62]	HPV causes 71% and 51.8% of all OPSCCs in the USA and UK, respectively

Keywords: HPV AND head and neck. *HNSCC* head and neck squamous cell carcinomas, *OPSCC* oropharyngeal squamous cell carcinomas, *RRP* recurrent respiratory papillomatosis

### HPV in andrological diseases

#### HPV-related lesions in the male

Since 2013, a high prevalence of HPV male genital tract infection has been reported, ranging from 50 to 70% [7]. Presence of HPV-DNA was reported in penile shaft, glans penis, coronal sulcus, semen as well as in scrotal, perianal, and anal regions. This finding suggested a possible role for males as reservoirs of HPV infection, which is favored by the evidence that HPV is a very resistant and ubiquitous virus [7].

In male patients, HPV infection has been associated, among others, with penile cancer [1]. Penile cancer is an aggressive and relatively rare squamous cell carcinoma of the skin of the glans or of the inner layer of the prepuce, characterized by invasive growth and early metastatic spread to lymph nodes [63, 64]. HPV is usually detected in one-third to one-half of penile cancers [65]. In particular, a recent study showed how HPV was responsible for 50.8% of penile cancers and for 79.8% of penile intraepithelial neoplasia [65]. Several different subtypes of HPV-related and non-HPV-related penile cancers have been described and, similar to HNSCC, they seem to have different prognostic profiles [66]. Moreover, as seen in HPV-related HNC, because of these two different pathogenic pathways, the new urological classification distinguishes between HPV-associated and non-HPV-associated penile carcinomas [64]. In Table 2, related articles published in the interval time January 2012–December 2022 are reported.

**Table 2:** Articles regarding HPV-related lesions in the male, published in English language, in the period January 2012–December 2022

References	HPV-associated diseases in men
[67, 68]	The two most common low-risk mucosal HPV subtypes are HPV-6 and 11, which together cause about 90% of genital warts
[68]	HPV-DNA is found in 47% to 48% of PCs, and most of these cases are caused by high-risk genotypes, preferentially HPV-16
[69]	HPV-related genital warts are mostly caused by HPV subtypes 6 and 11
[70]	Low-risk HPV types are associated with anogenital warts and the incidence and recurrence rates of anogenital warts are significantly increased in smokers
[71]	HPV-16 is present in about 80% of in anal carcinomas
[72]	Anogenital warts are caused mostly by low-risk human papillomaviruses such as HPV-6 and 11
[73]	Condylomata acuminata are caused by the two low-risk types HPV-6 and HPV-11 in more than 90% of cases
[74]	HPV might be associated with male genital lichen sclerosus
[75]	HPV might be associated with primary cutaneous adenosquamous carcinoma of the penis
[63]	The most common HPV subtype identified in PC is HPV-16, seen in 30.8%, while HPV-6 and HPV-18 represented 6.7% and 6.6%, respectively
[76]	Plantar warts are caused by HPV infection and most of plantar warts are caused by HPV-1
[77]	HPV has been previously evidenced in about 40% of cases of PC. In particular, HPV infection’s prevalence differed ranging from 22.4% in verrucous PC subtype to 66.3% for the basaloid/warty PC subtype
[78]	More than 90% of anal cancer cases are linked to HPV, especially with the high-risk HPV subtype (HPV-16)
[79]	HPV is detected in 30–50% of all invasive squamous PCs and HPV-16 is the most common subtype found
[80]	Genital warts and penile intraepithelial neoplasia might be caused by HPV; about 40% of PCs might be caused by HPV; HPV-related PCs may have a better prognosis than negative HPV-PCs
[81]	HPV-16 infection might represent a risk factor for the development of prostate cancer
[82]	Conflicting data about HPV vaccination and prevention of PC
[83]	Condyloma acuminata, anal and PCs might be caused by HPV
[3]	70% of anal and 60% penile cancer, genital warts
[84]	Anal intraepithelial neoplasia (AIN) and 89–100% of anal cancers are caused by persistent infections with high-risk HPV subtypes
[85]	78.4% of MSM has HPV infection (oral, anal, genital)
[86]	Prevalence of high-risk HPV (HR-HPV) in anal condylomas is 40.2% in immunocompromised and 16.4% in nonimmunocompromised patients. Moreover, HR-HPV in condylomas with high-grade squamous intraepithelial lesions (HSIL) is 73.8% and 17.7% in non-HSIL cases
[87]	About 50% of penile cancer
[66]	Penile squamous cell carcinoma
[88]	Penile squamous cell carcinoma
[89]	36% to 40% of penile cancer
[90]	About 40% of penile cancer
[91]	In MSM prevalence of anal, penile, oral and urethral HPV infection is 78.4%, 36.2%, 17.3% and 15.4%, respectively
[92]	HPV might play a role in bladder carcinogenesis and contribute to a worse prognosis for patients with bladder cancer

Key words: HPV and male. *PC* penile cancer, *MSM* men who have sex with men

**Fig. 2:**
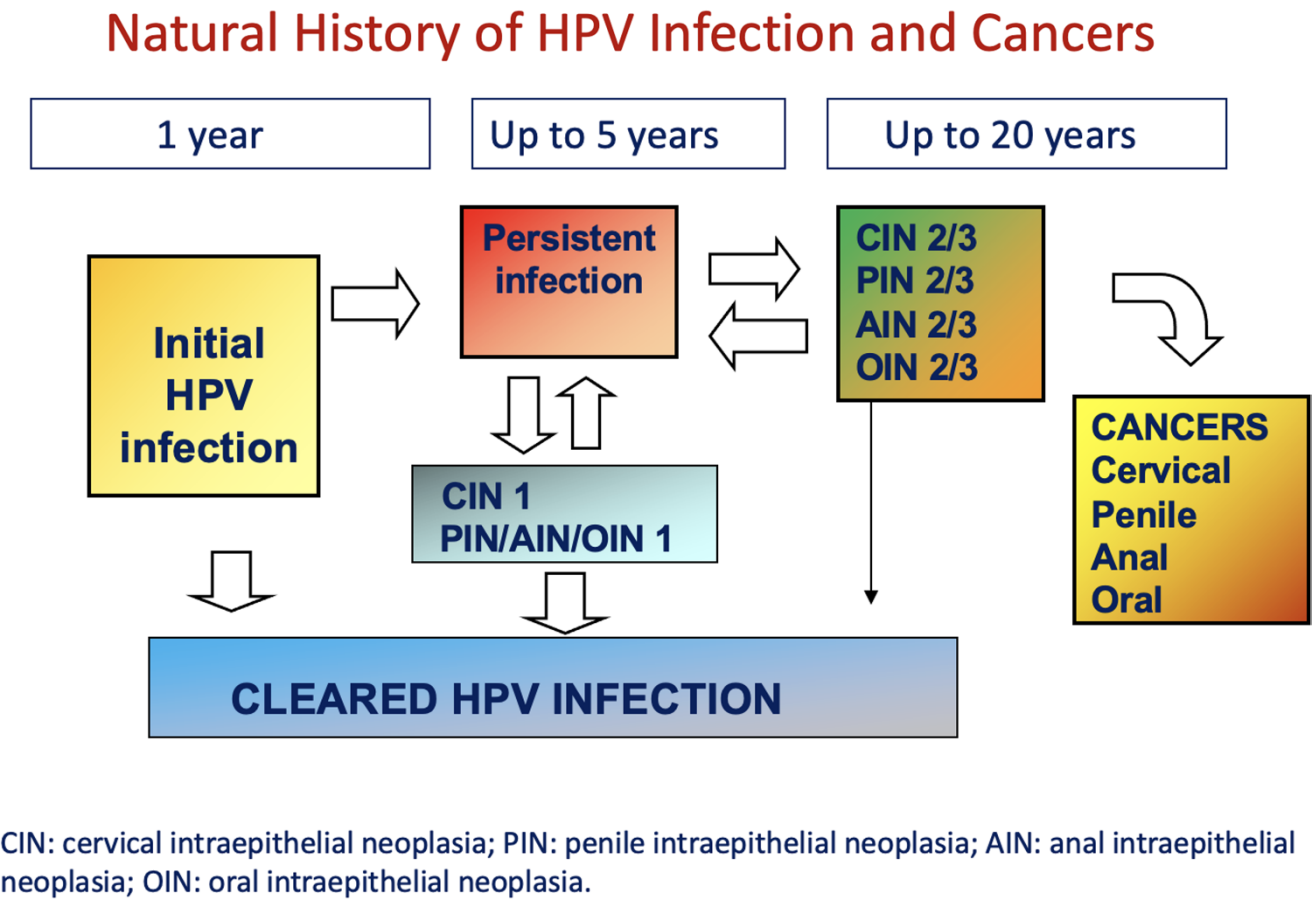
The parallelism between HPV infection and cervical intraepithelial neoplasia (CIN) and penile, anal and oral intraepithelial neoplasms (PIN, OIN and AIN)

These data appear to be noteworthy due to the possible parallelism between the natural history of HPV infection and cervical intraepithelial neoplasia (CIN), the precursor of cervical cancer [93] and the natural history of other well-known HPV-related neoplasms as penile intraepithelial neoplasia (PIN), anal intraepithelial neoplasia (AIN) and oral intraepithelial neoplasia (OIN) the precursors of penile, anal and oral cancers [94, 95] (Fig. 2).

#### HPV and male infertility

Infertility is defined as the failure to achieve a clinical pregnancy after 12 months or more of regular unprotected sexual intercourse [12]. Infertility affects 8–12% of couples globally, with a male factor being a primary or contributing cause in 50% of infertile couples [96].

During the last years, a great deal of interest has been drawn by the detection of HPV in semen. At this site, HPV has been detected both in exfoliated and sperm cells [1, 97]. Indeed, all the semen components (spermatozoa, somatic cells and/or plasma) may contain viral DNA [98]. Different semen samples from the same patient may test positive for HPV and more than one HPV genotype can be found in the same sample [98]. Such as already mentioned, all manuscripts evaluating the HPV prevalence in men affected by idiopathic infertility reported higher percentages of HPV infection compared with fertile controls [1]. HPV semen infection has been reported in about 2% of men from the general population and about 16% of men with unexplained infertility [99]. HPV-16 was the most common high-risk HPV found in the semen, whereas the second most common was HPV-56 [100]. Sepulveda et al. found, analyzing the current literature, a prevalence of seminal HPV infection in the general population of 8.2% and a prevalence of seminal HPV infection in the infertile population of 20.9% [101].

However, the negative impact of HPV semen infection on sperm parameters and male fertility seems to be independent from the detection of high-risk or low-risk HPV subtypes. In particular, HPV semen infection seems to be most related to asthenozoospermia and to anti-sperm antibodies (ASAs) [97, 102, 103].

Regarding sperm parameters, HPV has been associated to asthenozoospermia—defined as the percentage of progressively motile spermatozoa below the lower reference limit [104], reduced semen volume and count, increased DNA sperm fragmentation index ad altered pH and viscosity of the semen [105–107]. Moreover, HPV has been reported to affect the male fertility depending on the type of infected cells in semen [105].

It has been supposed that HPV presence on the sperm surface may represent a stimulus for ASA formation [108]. Regarding ASAs, their role in inducing male infertility is controversial [103]. Their levels seem to be higher in infected infertile patients compared to non-infected infertile men and the general population [105, 106]. Moreover, in infected infertile subjects, the presence of antibodies is associated with a further reduction of sperm motility, sperm agglutination, impaired cervical mucus penetration and interference with oocyte interaction [103, 106].

Similar findings have been found by Sepulveda et al. Their results indicated that HPV infection might cause detriment to seminal parameters, including a significant decrease in progressive motility and sperm morphology, and a significant increase in the sperm DNA fragmentation index when compared to HPV-negative patients [101].

Animal studies showed that fertility outcome is dependent on HPV genotype [109]. For instance, HPV-16 and 31 adversely impacted embryo development, whereas HPV-11, 16, 18 and 31 decreased the implantation rate [109]. Moreover, different in vitro experimental studies showed that HPV can negatively affect the very early embryo development and the trophoblastic cells invasiveness, suggesting a possible role of HPV in the reduction of implantation rate and pregnancy rate during ART (assisted reproductive technology) procedures [97, 110, 111]. Furthermore, HPV could play an additional negative role in male infertility, being often associated with other bacterial infections of male accessory glands (MAGI). In this light, some authors advocated the screening for HPV in patients with MAGI and testing semen for bacteria in HPV-infected infertile men [112]. The acknowledgment of all this evidence by the European Society of Human Reproduction and Embryology (ESHRE) has been formalized in 2021 through the release of the guidelines for medically assisted reproduction (MAR) in patients with viral infection or disease [113]. ESHRE guidelines recognize that HPV detection in semen is the only viral factor having a clinical association with assisted reproductive outcomes and suggest targeted counseling in infected couples undergoing MAR. So long that, some infertile couples cannot postpone the search for pregnancy and standard washing procedures used in ART are inadequate to reduce the seminal viral load**,** two possible strategies have been suggested in infertile couples with HPV semen infection: new strategies of sperm washing and HPV adjuvant vaccination. A previous study by our group showed the complete abrogation of HPV-DNA staining in positive sperm cells treated with heparinase III [114]. Nevertheless, this enzyme is not licensed for assisted reproduction in humans. Thereafter, virtual elimination of HPV adhering to the sperm surface was obtained through the application of a hyaluronidase-based sperm washing (IALu) procedure [109]. The enzyme hyaluronidase is approved for its use in in vitro fertilization (IVF) laboratories, and it is able to break the linkage between HPV and syndecan-like glycosaminoglycan components on the sperm surfaces [115]. The treatment of semen with IALu procedure could help to obtain the removal of HPV virions from sperm surface during ART, particularly in those cases where the natural immune process is expected to take a long time to clear HPV from semen [115]. In a recently published case series, two currently ongoing pregnancies, obtained after the application the IALu procedure in infertile couples with HPV semen infection, were reported [116].

Another possible strategy is represented by the adjuvant HPV vaccination. By this strategy, it was demonstrated that all infected patients achieved seroconversion after vaccination and, in a subset of them, a significant shortening of HPV time to clearance from semen was observed [117]. Therefore, HPV vaccination seems able to speed up the viral healing and to reduce the risk of viral recurrence [118]. De Toni et al. demonstrated that 86% of the patients receiving HPV vaccination obtained the complete clearance of HPV at both the seminal and genital level [97]. Moreover, they showed that the serum-antibody titer following vaccination was a sensitive marker of proper anti-HPV immune response [97]. A recent study showed how healing from HPV infection, obtained after HPV vaccination, was accompanied by an improvement of both the prevalence of anti-sperm antibodies and sperm motility [119]. Interestingly, vaccinated infertile couples who cleared HPV from semen had a better pregnancy rate than unvaccinated couples.

Testicular cancer is the most common solid tumor among males 15 to 34 years of age [120]. Well-established risk factors for testicular cancer include history of cryptorchidism, personal or family history of testicular cancer, age, and ethnicity [120]. The role of HPV in testicular cancer is controversial [10, 121]. Nevertheless, patients with testicular cancer have higher prevalence of HPV semen. Therefore, Garolla et al. suggested screening for HPV in testicular cancer patients at diagnosis and particularly after adjuvant treatments [121] because HPV infection can in turn induce cancer in many sites and reduce male fertility. In fact, adjuvant treatments—such as radio and chemotherapy—resulted strongly related to HPV infection susceptibility [121].

Here below we reported articles regarding HPV and male infertility, published in the period January 2012–December 2022 (Table 3).

**Table 3:** Articles regarding HPV and male infertility, published in the period January 2012–December 2022

References	HPV and male infertility
[122]	HPV is present in semen in 50% of patients with penile warts and in 41.3% of infertile patients
[123]	Demonstration of the presence of HPV gene sequences in Percoll-washed sperm cells
[124]	Incidence of asthenozoospermia among patients infected with either HPV is significantly higher than in those without HPV in their sperm cells
[125]	HPV is present in sperm cells from infected and apparently healthy subjects, and sperm washing does not eliminate HPV infection
[126]	In infected semen samples about 25% of sperm head an HPV-DNA positivity at the head site
[127]	A very high prevalence of infection in the semen is present in patients with risk factors for HPV (genital warts, infected partners, infertility). Sperm motility was reduced in infected samples
[128]	In couples undergoing ARTs, pregnancy loss rate correlates with positive HPV-DNA testing in the male partner
[129]	HPV can infect human sperm, it localizes at the equatorial region of the sperm head through interaction between the HPV capsid protein L1 and syndecan-1. Sperm transfected with HPV E6/E7 genes and sperm exposed to HPV L1 capsid protein are capable to penetrate the oocyte and transfer the virus into oocytes, in which viral genes are then activated and transcribed
[130]	Conventional sperm selection does not eliminate HPV sperm infection
[102]	HPV is present in 6.1% of the thawed cryovials in sperm banks
[114]	Modified swim-up by adding Heparinase III is able to completely remove HPV-DNA both from naturally and artificially infected sperm
[131]	7.8% of semen samples used for ART were HPV-DNA positive, but HPV infection did not seem to affect semen quality. HPV is localized at the equatorial region of the sperm head
[103]	Infertile patients with HPV semen infection show high percentages of ASAs. In these patients, HPV sperm infection is associated with lower sperm motility, which is worse in subjects with ASAs. To obtain a significant clearance of both HPV sperm infection and ASAs, at least 24 months of follow-up are needed
[132]	Infertile males have a relatively high HPV infection rate compared with fertile males. Sperm progressive motility and the normal morphology rate are significantly decreased in HPV-positive subjects. HPV-45, HPV-52, HPV-18, HPV-59 and HPV-16 infections are more frequently in infertile males
[133]	Sperm DNA fragmentation index is not increased in semen containing HPV
[112]	Patients with Male Accessory Gland Infection (MAGI) had a significantly higher frequency of HPV infection compared with controls; patients with MAGI and HPV had a significantly lower sperm progressive motility and normal morphology compared with patients with MAGI HPV-negative
[119]	Humoral immunity has a major role in healing from HPV infection Vaccinated patients showed improved healing, achieving clearance in 12 months
[111]	A reduction in natural and assisted cumulative pregnancy rate and an increase in miscarriage rate are related to the presence of HPV at sperm level
[134]	Adjuvant vaccination is associated with enhanced HPV healing in semen cells and increased rate of natural pregnancies and live births
[135]	The seminal infection by high-risk HPV is associated with impaired sperm progressive motility and a higher sperm DNA fragmentation index
[136]	Semen volume, sperm count, sperm motility and the normal morphology rate are significantly decreased in HPV-positive. Sperm motility is significantly decreased in ASA-positive subjects and patients with HPV infection have a higher rate of ASA than the non-HPV group
[137]	In infected sperm cells, L1 is co-localized with aquaporin 8 (AQP8). HPV infection might directly inhibit AQP8 functionality thus making sperm cells more sensitive to oxidative stress
[97]	HPV adjuvant vaccination in males with genital tract infection induces the clearance of genital HPV-DNA in 86% patients. A serum-antibody titer at 6 months after vaccination equal to or greater than the threshold value 1:125 is prognostic of healing
[101]	Infertile men have a higher prevalence of seminal HPV infection compared to the general population, regardless of the HPV genotype detected. HPV infection is related to a significant decrease in progressive motility, a low sperm morphology score, and a significant increase in the sperm DNA fragmentation index. Patients undergoing ART with seminal HPV infection have an increased risk of miscarriage and a reduced chance of ongoing pregnancy
[105]	HPV-positive semen samples exhibited differences in the taxonomic composition of the bacterial microbiota including higher abundances of Moraxellaceae, Streptococcus and Peptostreptococcus
[115]	The treatment of semen samples with hyaluronidase is associated with the complete loss of HPV-DNA signal
[138]	HPV and Chlamydia trachomatis infections are reciprocal risk factors of their co-infection in males
[139]	No differences in sperm conventional parameters are reported in patients infected by low-risk HPV than in controls
[140]	The prevalence of HPV sperm infection was significantly higher (25%) in couples affected by recurrent pregnancy loss than in their fertile counterparts
[116]	The treatment of semen samples with hyaluronidase (IALu procedure) is an effective approach for straightforward fertility treatments in cases of recurrent HPV-DNA semen detection

Keywords: HPV and male infertility. *ASAs* anti-sperm antibodies, *ARTs* assisted reproductive technologies

#### Therapies and future perspectives

It has been reported that the majority of HPV-related cancers are preventable through HPV vaccine, when administered before exposure [4]. Currently, there are 3 different HPV vaccines that have been, or are currently being used to prevent HPV-related cancers worldwide: the bivalent HPV vaccine (Cervarix, by GlaxoSmithKline), the quadrivalent HPV (4vHPV) vaccine (Gardasil by Merck) and the nonavalent HPV (9vHPV) vaccine (Gardasil-9 by Merck) [4].

All three available prophylactic vaccines show high efficacy in prevention of vaccine-specific HPV-type infection, with the highest degree of protection achieved in the population of HPV-naive women [141, 142]. Actually, 9vHPV—protecting against HPV types 6, 11, 16, 18, 31, 33, 45, 52, 58—is routinely recommended to both males and females from ages 9 to 45 years of age to help prevent dysplastic lesions caused by human papillomavirus [143]. Moreover, recent data suggest that vaccination speed up viral HPV clearance in infected patients [117, 118].

During the last years, therapeutic HPV vaccines, capable of generating T cell-mediated immunity against HPV infection and associated diseases, have been developed [144, 145]. They include live-vector, protein, peptide, dendritic cell, and DNA-based vaccines [144]. However, these therapeutic HPV vaccines are still being developed and are not allowed for clinical use. The only recently tested therapeutic vaccine is VTP-200, which is currently being experimented in a phase I/II placebo-controlled trial, against low-grade cervical lesions [145].

While HPV vaccines are very effective in preventing infection, there are still problems regarding HPV vaccination: (i) guidelines suggest screening for related cancers in vaccinated persons [142]; (ii) disparities in HPV vaccinations are present and frequent worldwide [5]; (iii) adolescents and young adults are poorly informed about HPV and the vaccination issues, underestimating the likelihood of HPV infection [146–148] and in particular, a recent study evaluating HPV knowledge in 1000 young Italian males, reported that only about half of the participants had heard about HPV infection and only 58% of the overall males reported that they would be willing to receive the HPV vaccine [149]; (iv) medical students are poorly informed too [150]; (v) recent evidence showed that awareness of HPV and its vaccine is declining in general population [151]. An overwhelming role in information about HPV could be supplied by social media, representing a source of health information [152]. In conclusion, albeit there is an increase in the awareness of the HPV vaccination campaign among the population, a greater effort might be required, and even mandatory, in order to enhance the awareness of HPV infection in males and in the young population. In addition, regarding male patients, medical specialists should evaluate the sexual practices of all male patients, especially men who have sex with other men, and educate them on the HPV infection risks, especially in the light of the evidence that anal cancer among men who have sex with men was found to be more common than cervical cancer among women [153].

## Conclusion

HPV is responsible for oncological diseases both in males and in females. Furthermore, HPV infection is able to induce male infertility and it is associated with early miscarriages. Therefore, HPV infection represents a health problem with a detrimental social and public impact. Despite this evidence, little has been done to date to widely promote vaccination among young males. Therefore, it is to be hoped that more efforts will be made in the future to promote male vaccination campaigns and to better define the “higher risk” male populations to be screened for HPV infection and possibly to be vaccinated. Finally, further studies are needed both to clarify the efficacy of sperm washing procedures on the results of assisted fertilization techniques, and to shed light on the clinical impact of HPV vaccination in terms of improvement of natural fertility.
